# Asymmetric Arc Routing by Coordinating a Truck and Multiple Drones

**DOI:** 10.3390/s22166077

**Published:** 2022-08-14

**Authors:** Shuangxi Tian, Honghui Chen, Guohua Wu, Jiaqi Cheng

**Affiliations:** 1 College of Systems Engineering, National University of Defense Technology, Changsha 410073, China; 2 College of Business Administration, Hunan University of Finance and Economics, Changsha 410205, China; 3 School of Traffic and Transportation Engineering, Central South University, Changsha 410075, China

**Keywords:** truck-drones arc routing, traffic patrol, large-scale neighborhood search

## Abstract

Unmanned Aerial Vehicles, commonly known as drones, have been widely used in transmission line inspection and traffic patrolling due to their flexibility and environmental adaptability. To take advantage of drones and overcome their limited endurance, the patrolling tasks are parallelized by concurrently dispatching the drones from a truck which travels on the road network to the nearby task arc. The road network considered in previous research is undirected; however, in reality, the road network usually contains unidirectional arcs, i.e., the road network is asymmetric. Hence, we propose an asymmetric coordinated vehicle-drones arc routing mode for traffic patrolling. In this mode, a truck travelling on an asymmetric road network with multiple drones needs to patrol multiple task arcs, and the drones can be launched and recovered at certain nodes on the truck route, making it possible for drones and the truck to patrol the task in parallel. The total patrol time is the objective function that needs to be minimized given the time limit constraints of drones. The whole problem can be considered as an asymmetric arc routing problem of coordinating a truck and multiple drones. To solve this problem, a large-scale neighborhood search with simulated annealing algorithm (LNS-SA) is proposed. Finally, extensive computation experiments and a real case are carried out. The experimental results show the efficiency of the proposed algorithm. Moreover, a detailed sensitivity analysis is performed on several drone-parameters of interest.

## 1. Introduction

With the rapid growth of car ownership, traffic congestion has become a severe problem in metropolises [1]. To reduce the congestion, information on the traffic conditions plays a vital role [2]. At present, traffic information is obtained through traditional police patrols by ground vehicles [3] or through static sensors such as digital cameras [4]. These approaches lack flexibility and are restricted to the road network. For example, the static sensors can only detect fixed areas, and when the roads are heavily congested, the patrol vehicle can hardly enter the road to gain traffic information. In recent years, Unmanned Aerial Vehicles, knowns as drones, have drawn considerable attention due to their environmental adaptability, flexibility, and potential uses in logistics and monitoring [5]. Drones can carry different types of loads to perform different kinds of tasks, such as line inspection and traffic patrolling. However, the widespread application of drones is limited by their battery capacities [6]. One solution to overcoming the drones’ disadvantage is to coordinate ground vehicles and drones.

Coordination between ground vehicles and drones could combine their advantages to enhance efficiency [7]. In detail, the vehicle acts as a charging platform for drones to make up for their limited flight endurance [8]. The combination of vehicle and drones has been applied to parcel deliveries [9]. For example, Murray et al. [7] considered a vehicle and a drone for the last mail delivery, and proposed the flying sidekick traveling salesman problem (FSTSP). Karak et al. [10] presented a mathematical formulation for the hybrid vehicle-drone routing problem (HVDRP) for pick-up and delivery services. The researchers developed an extended Clark and Wright algorithm to solve the HVDRP. Furthermore, other methods such as the large neighborhood search (ALNS) [11,12], tabu search with simulated annealing algorithm (TS-SA) [13], and variable neighborhood search (VNS) [14] were used to solve the coordinated vehicle-drone planning problem. It can be concluded that previous works are mainly oriented to parcel deliveries which point targets abstractly. This paper focuses on the patrol routing problem on an urban road network, which is actually a line task. This problem can be defined as a coordinated vehicle-drone arc routing problem [15].

At present, the coordinated vehicle-drone arc routing problem has motivated a few optimization approaches with the objective of reducing time or cost. Among the available studies, Liu et al. [16] investigated a high-voltage power line inspection system with a ground vehicle and a drone. To optimize the route of vehicle and drone, the researchers constructed a two-layer heuristic to solve such problem. Luo et al. [17] first proposed a traffic patrol mode consisting of a ground vehicle and a drone in an urban road system; both arc tasks and point tasks are considered in their traffic patrol mode, and they presented a two-stage heuristic approach to scheduling the ground vehicle and the drone. In the scenarios of the above two studies, only one vehicle and one drone are considered, which is unrealistic considering that a ground vehicle can carry more than one drone. Therefore, Wu et al. [18] focused on the scheduling problem with one vehicle and multiple drones, which is closer to reality and more difficult to solve. An adaptive large neighborhood search algorithm was developed to solve the problem. In their work, the asymmetry of the road network was not considered, while one-way roads in the road network are more likely to be congested. Hence, this study focuses on the asymmetric coordinated vehicle-drones arc routing problem (A-VD-ARP) which means some arcs of the road network in this problem are unidirectional.

This paper studies an extension of the arc routing problem (ARP) where a truck is collaborating with multiple drones. Since the problem is a generalization of the classic ARP, it is NP-hard to solve. It is a great challenge to solve A-VD-ARP due to the fact that arc tasks have a dimension of direction compared with point tasks. In this article, a metaheuristic method of large neighborhood search with simulated annealing (LNS-SA) algorithm is proposed to solve the coordinated planning problem discussed above. Under the LNS-SA framework, an initial solution is generated through a heuristic method, and then we design several neighborhood operators based on the characteristics of the A-VD-ARP to optimize the solution and expand the search space. Experiments demonstrate that the proposed method can significantly improve the efficiency of coordinated vehicle-drones planning.

The main contributions of this paper are summarized as follows.

(1) For the first time, we investigate the coordinated vehicle-drone arc routing problem under an asymmetric road network, which is closer to reality. In this scenario, a truck, which carries multiple drones, departs from the depot, travels on the road network and returns to the depot after patrolling all the tasks. Drones take off from the truck, patrol one or several arc tasks, and return to the truck.
(2) In order to solve the above problem, we propose a metaheuristic method with an innovative coding scheme, a heuristic method for obtaining the initial solution and six neighborhood operators for improving solutions. Through the LNS-SA framework, the solution is iteratively optimized.
(3) We conduct extensive experiments on simulated and realistic scenarios to verify the effectiveness of the proposed method. We design a total of 9 experimental scenarios on 3 datasets and a realistic scenario on Changsha City. The proposed algorithm is compared with simulated annealing (SA), variable neighborhood search (VNS), and tabu search (TL). The computational results clearly prove that the proposed algorithm can obtain a satisfactory solution in acceptable time.

The structure of this paper is as follows. In Section 2, we summarize the research of related works. In Section 3, we describe the A-VD-ARP and construct a mathematical model. In Section 4, a large-scale neighborhood search framework combined with a simulated annealing mechanism is proposed, and the corresponding neighborhood structure is given according to the characteristics of the problem. Section 5 summarizes and discusses the experimental results. Finally, Section 6 concludes the full text and presents the future research direction.

## 2. Related Work

The coordinated vehicle-drone arc routing problem has a wide range of application scenarios in real life. In the arc routing problem, most researchers do not consider the cooperation between vehicles and drones. There are few studies on the coordinated vehicle-drone arc routing problem. We analyze the coordinated vehicle-drone arc routing problem from three aspects: (1) the arc routing problem, (2) point-target-oriented vehicle-drone coordination, and (3) the coordinated vehicle-drone arc routing problem.

In the arc routing problem, there are three important branches: the Chinese Postman Problem (CPP) [19,20], the Rural Postman Problem (RPP) [21,22,23], and the arc routing with capacity constraints problem (Capacitated Arc Routing Problem, CARP) [24,25,26,27]. For the Chinese Postman Problem (CPP), Nilofer et al. [20] considered passing as many important nodes as possible while making the vehicle traverse at least the edges in the graph. The Rural Postal Route Problem (RPP) only visits some of the edges in the graph, which is the general form of the CPP problem. Calogiuri et al. [21] proposed a branch and bound algorithm to solve the RPP exactly. Monroy-Licht et al. [22] used a large-scale neighborhood search algorithm to solve the problem of rural postal route with time windows. This algorithm can quickly and efficiently shorten the calculation time in large-scale instances and obtain satisfactory results. The capacity-constrained arc routing problem (CARP) takes into account the vehicle’s maximum range limit. Huang et al. [24] used the ant colony algorithm to solve the CARP problem with time windows. Xing et al. [25] proposed an extended multi-park capacity-constrained arc routing problem (MCARP), which was optimized by an evolutionary algorithm. Besides tabu search [28], hybrid heuristic algorithm [29] and divide and conquer algorithm [30] are also used to solve the CARP problem.

In the research on vehicle-drone collaboration, Murray et al. [7] proposed the Flight Assisted Traveling Salesman Problem (FSTSP) and designed a heuristic algorithm to solve it. Agatz et al. [31] proposed the Drones Traveling Salesman Problem (TSP-D) and designed a “path first, cluster second” heuristic algorithm to solve it. For the TSP-D model, Ha et al. [32] first generated the TSP path, then decomposed the TSP-D path from the TSP path, and then optimized the TSP-D path using the LS operator. Tu et al. [33] proposed an adaptive large neighborhood search algorithm to solve the TSP-mD model containing multiple drones. Hu et al. [34] proposed an algorithm that first used a K-means clustering approach to find a reasonable launch position for the drone, and then determined the route of the ground vehicle based on the genetic algorithm. Pan et al. [35] presents an innovative schedule approach by coordinating the logistic drone and crowdsourced buses. In order to reduce solution time, Wu [36] proposed a reinforcement learning approach to solve the truck-and-drone coordinated delivery problem efficiently. Except for package delivery, Huang et al. focused on the deployment of a charging station for aerial surveillance by drones. Trotta et al. [37] proposed a bus-drone coordinated surveillance mode. Tian [38] presented a target surveillance mode by coordinating a truck and multiple drones. The researchers proposed a new heuristic method to optimize the truck and drone routes. In addition, Dorling et al. [6] and Wang et al. [39] extended the vehicle-drone coordination problem from single-vehicle and multi-drone to multi-vehicle and multi-drone.

For the coordinated vehicle-drone arc routing problem, Liu et al. [16] proposed a two-layer point-arc routing problem model to cooperate with ground vehicles and drones for high-voltage power lines inspection; they designed two heuristic algorithms of “cluster first, then sort” and “sort first, then decompose” to solve the problem. Luo et al. [17] proposed a traffic patrol model for heterogeneous tasks in urban road systems, in which ground vehicles only serve as the charging platform for the drones, and drones perform point and arc tasks along the road network. Wu et al. [18] consider the scheduling problem with one vehicle and multiple drones, and propose a metaheuristic method to solve the problem efficiently.

According to the above review, it can be concluded that the traditional arc routing problem has been extensively studied, and, secondly, in the field of vehicle-drone coordination, the research on point targets has also been widely investigated. But the research on the vehicle-drone cooperation problem oriented to line targets is still missing, while the coordinated vehicle-drone arc routing problem has a wide range of application scenarios in real life. Asymmetric arc routing by coordinating a truck and multiple drones is considered in this article, and a metaheuristic algorithm is proposed to solve this problem.

## 3. Problem Description

The proposed A-VD-ARP in this article is the following: a truck with multiple drones departs from the patrol center, travels on the asymmetric road network—which means some arcs of the road network are unidirectional—and returns to the patrol center after patrolling all the arcs in the road network. The drones can be launched and recovered from the truck at the node (intersection) of the road network, and the drones can access one or several target arcs at one time as long as the maximum flight time is not exceeded. Both the truck and the drones can perform the patrol task. Considering the heavily congested road which cannot be patrolled by the truck, some of the target arcs are required to be accessed only by drones. In addition, the truck must travel along the road network, while the drones are not restricted to the road network. To simplify the problem, the time of launching/recovering a drone is incorporated into the travel time as in references [16,31].

**Figure 1:**
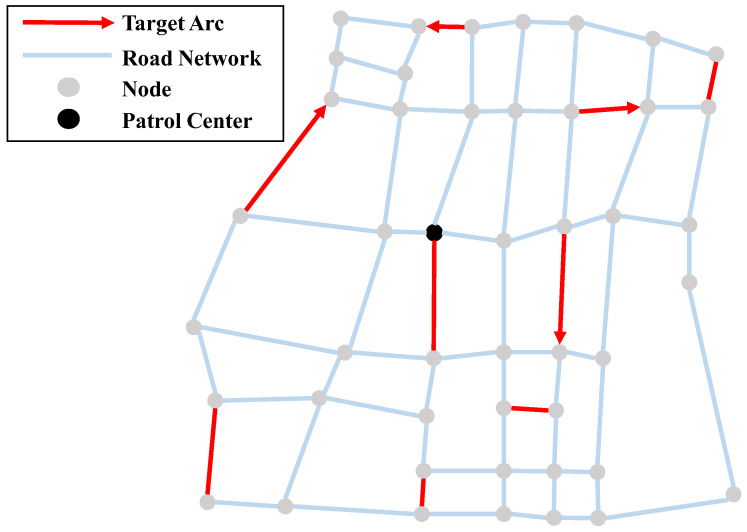
Schematic diagram of the road network and arc tasks.

The asymmetric road network in this paper is simplified as a directed connected graph, represented by G=(V,A). The set of road intersections is the point set V={1,2,…,n}, where n represents the number of intersections in the road network. The set of edges is represented as $A=\left\{a_{ij}=\left(i,j\right)|i,j\in V\right\}$, where arc $a_{ij}$ connects node i and node j. Each arc has a non-negative weight $w_{ij}$, which represents the length of the edge. If $a_{ij}\notin A$, then $w_{ij}=\infty$. Moreover, since the road network is asymmetric in this article, so $a_{ij}\neq a_{ji}$. We define the set of general target arcs as T. The set of target arcs that can only be performed by drones is presented as TD. Figure 1 shows an example scenario in this article. In addition, other symbols and descriptions are shown in Table 1.

### 3.1. Objective Function

The objective function of the A-VD-ARP is to minimize the total time for the truck and drones to coordinately perform all patrol tasks and return to the depot. Let $t_{PC}$ and $t_{PC}^{k}\;$ denote the time when the truck and the k-th drone return to the patrol center, respectively. Then the objective function can be expressed as:

(1) $$\min\max\left\{t_{PC},\;t_{PC}^{k}\right\}$$

### 3.2. Vehicle Route Constraints

Let the binary variable $x_{ij}$ denote whether the truck patrols the task arc $a_{ij}$. If the truck accesses the arc $a_{ij}$, $x_{ij}=1$; otherwise, $x_{ij}=0$. The following constraints should be satisfied for the truck.

(2) $$\underset{a_{ik}\in A}{\sum }x_{i,k}=\underset{a_{kj}\in A}{\sum }x_{k,j},\forall k\in V$$

(3) $$\underset{a_{0,i}\in A}{\sum }x_{0,i}\geq 1,\forall i\in V^{+}$$

(4) $$\underset{a_{j,PC}\in A}{\sum }x_{j,PC}\geq 1,\forall j\in V^{-}$$

Constraint (2) guarantees the continuity of the truck route. Constraints (3) and (4) ensure that the truck departs from the patrol center and eventually returns to the patrol center.

### 3.3. Drone Route Constraints

Let the binary variable $y_{s,e}^{k}$ denote that the k-th drone is released from node s and recovered at node e. For the access path of the drone, there are the following constraints:

(5) $$\underset{e\in V}{\sum }y_{s,e}^{k}=1,\forall s\in V,k\in D,a_{s,e}\in A$$

(6) $$\underset{s\in V}{\sum }y_{s,e}^{k}=1,\forall e\in V,k\in D,a_{s,e}\in A$$

Constraints (5) and (6) ensure that each flight route of the drone has only one release node and one recovery node.

(7) $$\underset{j\in V}{\sum }y_{i,j}^{k}\leq \underset{s\in V}{\sum }x_{i,s},\forall i,j\in V,k\in D,j\neq s$$

(8) $$\underset{i\in V}{\sum }y_{i,j}^{k}\leq \underset{v\in V}{\sum }x_{v,j},\forall i,j\in V,k\in D,i\neq v$$

Constraints (7) and (8) respectively ensure that the release and recover nodes for the drones must be the nodes on the truck route, that is, they ensure the cooperative relationship between truck and drones.

The launch and recover order of the drones must also be constrained. A drone can be released only if it has never been released or has been recovered since the last release, otherwise, it cannot be released at node i.

For the truck, when reaching a launching node, at least one drone needs to be on the truck.

(9) $$av_{i}^{k}\left(\underset{j\in V^{+}}{\sum }y_{i,j}^{k}\right)=0,\forall i\in V,a_{ij}\in A$$

(10) $$x_{i,j}\left(\underset{v\in V^{-}}{\sum }y_{v,j}^{k}\right)av_{j}^{k}=0,\forall i,j\in V,a_{ij}\in A$$

(11) $$\underset{k\in D}{\sum }\left(av^{t,k}\right)\leq d$$

Constraints (9) and (10) stipulate that only the drones on the truck can be launched and only the drones that have been released can be recovered. Constraint (11) ensures that at most d drones are in flight at any time.

Due to the limitation of the battery capacity of the drone, each drone has a maximum flight time during one flight. Assuming that the maximum flight time of the drone is P, then for each drone path $\langle s,ce_{i},e\rangle$, the following constraints should be satisfied:

(12) $$t_{e}^{k}-t_{s}^{k}\leq P$$

(13) $$t_{e}-t_{s}^{k}\leq P$$

### 3.4. Time Constraints and Task Constraints

The time constraints for the truck and drones are as follows:

(14) $$t_{j}\geq t_{i}+\frac{w_{ij}}{v_{g}}-M\left(1-x_{i,j}\right),\forall i,j\in V,i\neq j$$

(15) $$t_{j}^{k}\geq t_{i}^{k}+\frac{w_{ij}}{v_{d}}-M\left(1-y_{i,j}^{k}\right),\forall i,j\in V,i\neq j$$

Constraints (14) and (15) represent the earliest arrival times of the truck and drones at each node, respectively, where M is an infinite positive number.

Each task needs to be performed by the drone or the truck at least once, subject to the following constraints:

(16) $$x_{ij}+\underset{k}{\sum }\left(y_{i,j}^{k}+y_{j,i}^{k}\right)\geq 1,\forall a_{ij}\in T$$

(17) $$\underset{k}{\sum }\left(y_{i,j}^{k}+y_{j,i}^{k}\right)\geq 1,\forall a_{ij}\in TD$$

## 4. Solution Method

According to the characteristic of the A-VD-ARP, we designed a metaheuristic based on large-scale neighborhood search and simulated annealing mechanism (LNS-SA). First, the initial solution is constructed by a heuristic method, then, neighborhood search strategies are applied under the LNS-SA framework to iteratively optimize the solution.

The large-scale neighborhood search was introduced by Shaw in 1998 [40]. The solution is searched by the destruction operator and the repair operator, which greatly enhances the search ability of the algorithm. Simulated annealing was proposed by Kirkpatrick in 1983 [41], which can effectively avoid falling into a local minimum.

In this paper, the large-scale neighborhood search and simulated annealing are combined to improve the efficiency of the algorithm. The LNS-SA algorithm framework is shown in Algorithm 1. Among them, the initial solution is obtained by the heuristic algorithm described in Section 4.1, and the neighborhood list contains a certain number of neighborhood search strategies. The algorithm first initializes the solution, the current solution, the current temperature, and the current number of iterations (Line 2). Then, in the main loop of the LNS-SA algorithm (lines 3–19), the neighborhood structure is selected to adjust the current solution until the termination condition is satisfied. Whether the obtained solution is accepted depends on the simulated annealing criterion (lines 7–11). Each time the main loop is executed, the iteration count and termination temperature are updated (Lines 4, 19).
**Algorithm 1:** Framework of LNS-SA
**Input:** Initial temperature
$T_{0}$; termination temperature
$T_{min}$; maximum iteration number
$I_{max}$; annealing rate
γ; initial solution
$s_{0}$; destruction operator collection
D; repair operator collection
R

**Output:** Optimal solution
s
1**Get**$s_{0}$ through the heuristic method2**Initialize** 
$s\leftarrow s_{0}$
$;\;s^{\prime }\leftarrow s_{0}$
$;\;T\leftarrow T_{0}$
; iter←0
3**While** $\left(iter<I_{max}\right)$$\;\mathrm{and}\;(T>T_{min})$  **do**4
    iter←iter+1
5
    Random select destroy operator d∈D
 and repair operator r∈R
6
    $s^{*}\leftarrow r\left(d\left(s^{\prime }\right)\right)$
7**If** 
$f\left(s^{*}\right)<f\left(s^{\prime }\right)$ **then**
8
         $s^{\prime }\leftarrow s^{*}$
9**If**$f\left(s^{*}\right)<f\left(s\right)$ **then**10
           $s\leftarrow s^{*}$
11**End if**12**Else**13
    $\Delta f_{1}\leftarrow \left(f\left(s^{\prime }\right)-f\left(s\right)\right)$
14
        $\Delta f_{2}\leftarrow \left(rand\left(1\right)<\exp\left(-\Delta f_{1}/T\right)\right)$
15     **If** exp(Δf/T)>ζ, where ζ←uniform(0, 1)  **then**16
          $s\leftarrow s^{\prime }$
17          
**Break**
18
     **End if**
19
    T=γT
20**End while**

### 4.1. Initial Solution Generation

For neighborhood search algorithms, the quality of the initial solution often plays an important role in the final optimization result and the performance of the algorithms. In the construction of the initial solution, we firstly assign arc tasks to drones or the truck under the condition that the constraints are satisfied. The access direction of each task arc is determined under the road network constraint considering the road network is asymmetric. Notice that the drone is not restricted to the road network, so the access direction for drones can be randomly determined. For the task arcs that are visited by drones, allocate the launch and recovery nodes that satisfies the drones’ flight constraints; after doing this, each route of the drone can be uniquely determined. Finally, the route of the truck is generated by inserting the drone launch and recovery node sequence without violation of the maximum flight time of the drone, and afterwards, all tasks performed by the truck are inserted into the truck route. At this point, an initial solution of the arc routing scheme for single-truck and multiple drones is formed. The process of preprocessing the road network and generating the initial solution is shown in Algorithm 2.
**Algorithm 2:** Heuristic method for initializing solution
**Input:** Road network
G

**Output:** Initial solution
s
1**Initialize** The truck route
$s_{gv}\leftarrow \;[]$, the
k-th drone’s route
$s_{d}^{k}\leftarrow \;[]$
2Calculate the shortest distance between any two nodes by Floyd algorithm3**While**T≠∅  **do**4
   **If** $task_{i}\in TD$
5       Randomly select a pair of take-off and landing nodes and the access direction6Insert the take-off and landing nodes in the truck route.7       Delete $task_{i}$ from 
T
8   **else**
9       Insert the two endpoints of the task to the truck route10       Delete 
$task_{i}$ from 
T
11   **End if**
12**End while**

**Figure 2:**
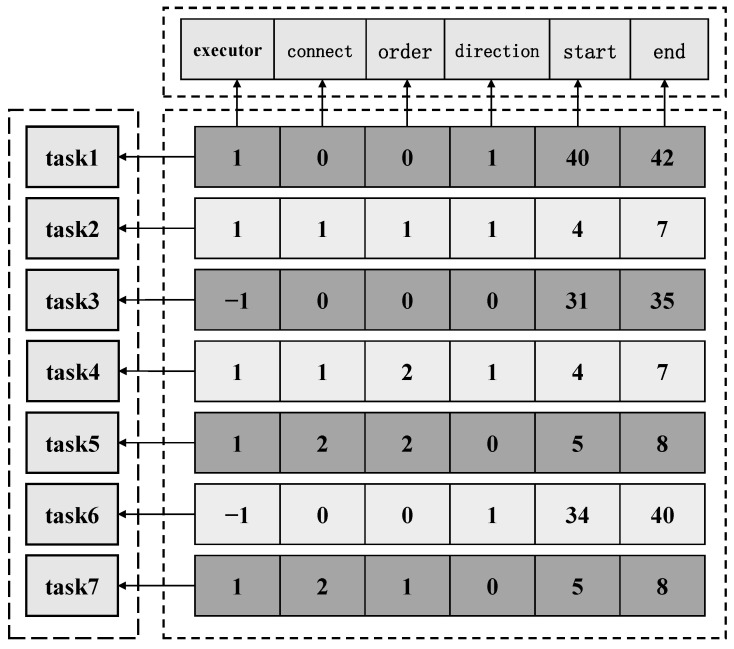
The Encoding of variables.

Specifically, we encode the route information of the truck and the drones into two matrices, task_infor and vehicle_path. The encoding information of the two matrices is described below.

task_infor is a matrix of size n×6 used to store task arc information, where n is the number of task arcs. As shown in Figure 2, each row of the matrix stores one task arc information. The first column stores the executor of the task arc. If it is 1, it means that the task arc is accessed by the drone, and −1 means that the task arc is accessed by the truck. The second column stores the information about whether the task arc belongs to a linked task. 0 indicates that the task arc is an independent task arc. Numbers such as 1, 2, … represent the linked task, and task arcs in the same linked task are represented by the same number. The numbers in the third column indicate the access order of the arcs in the linked task. If the task is an independent task, the number in the third column is 0. If the arc is in a linked task, the number in the third column is a natural number greater than 0 whose size indicates the order of access. The fourth column stores the access direction of the task arc, 0 means forward, 1 means reverse. The fifth and sixth columns store the start and end nodes of the task arc, respectively. If the task edge is assigned to the truck, the start node and the end node are the two endpoints of the task arc. If the task edge is assigned to a drone, one pair of take-off and landing point pairs that satisfies the drones’ flight time constraint is stored; all the arcs in a linked task use the same take-off and landing node pair.

The size of the vehicle_path matrix is 2×N. The first row stores the nodes that the truck needs to visit. These nodes are obtained from the last two columns of the task_infor matrix. The second row stores the identifiers of the tasks. The route of the truck and drones can be uniquely determined by the above two matrices.

### 4.2. Neighborhood Structure Design

The neighborhood structures designed in this paper are composed of a destroy operator (DO) and a repair operator (RO) in pairs. According to the characteristics of A-VD-ARP, we design a total of 6 destroy operators and 1 repair operator. The destroy operators mainly change the assignment, execution direction, and connection of task arcs, and delete the corresponding nodes in the truck route. And the repair operator is to re-insert the nodes deleted by the destroy operator into the truck route.

The specific operation process of each destroy operator and repair operator will be introduced below, and the schematic diagram will be used for visual representation. In the schematic diagram of all operation changes, the solid/dotted line between two nodes does not represent an edge in the road network but the shortest route between two nodes in the road network (we calculated the shortest path between any two nodes in the road network in advance using the Floyd algorithm). The solid line represents the truck route, the dotted line represents the drones’ routes, and the solid nodes represent the nodes involved in the operation of the selected destroy operator/repair operator.

#### 4.2.1. Destroy Operator

The operations of the destroy operator mainly include randomly selecting a task arc, changing its access direction, the assignment of the task (drone or truck), the pair of take-off and landing nodes, the connection with other arcs, and removing it from the truck route.

(1) Change access direction of the task

**Figure 3:**
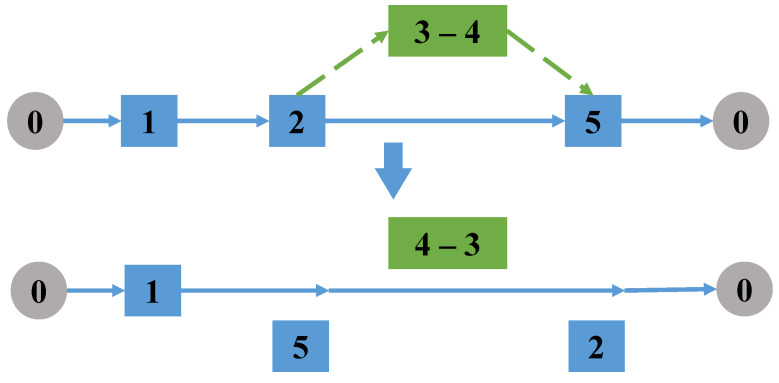
Change the direction of the tasks.

Randomly select a task arc accessed by the drone. If it is a single task accessed by a drone, delete the take-off and landing node of the task arc from the truck route, and exchange the take-off and landing node pair to change the access direction of the task; if it is a linked task, all tasks in the linked task are reversed, the take-off and landing points of the linked task are exchanged, and then delete the node pair from the truck route, as shown in Figure 3.

(2) Change the take-off and landing point

**Figure 4:**
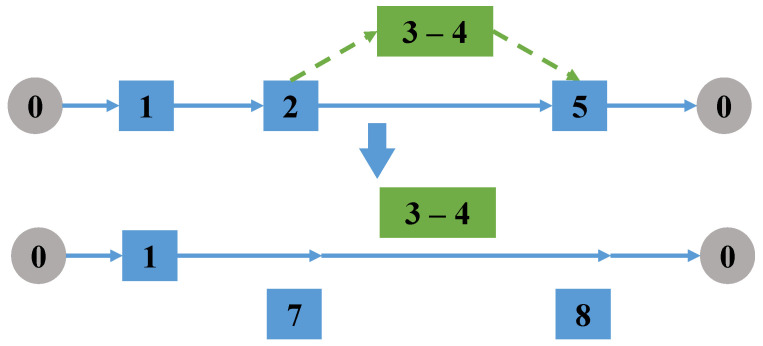
Change the takeoff and landing points of the task.

Randomly select a task performed by a drone (a single task arc or a linked task that contains subtasks), delete the take-off and landing node pair of the selected task in the truck route. Then, randomly re-select a pair of take-off and landing nodes in the set of take-off and landing node pairs of the task, as shown in Figure 4.

(3) Update the truck route

**Figure 5:**
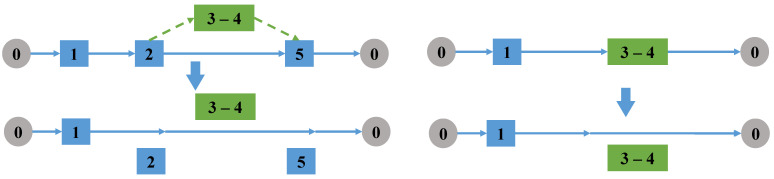
Change the route of the vehicle.

Randomly select a task, if it is a task performed by a drone, delete the take-off and landing nodes of the drone task from the truck route; if it is a task performed by the truck, directly delete the two endpoints of the task from the truck route, as shown in Figure 5.

(4) Change the assignment of a single task

**Figure 6:**
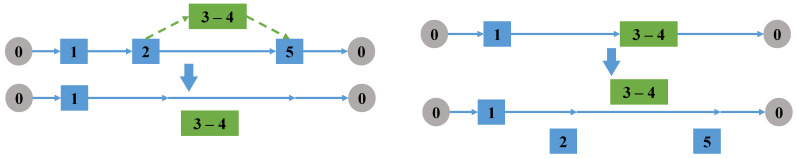
Change the assignment the task.

Randomly select a single task arc. If the selected task is performed by a drone, determine whether the task can be assigned to the truck. If so, delete the take-off and landing nodes from the truck route and re-assign the task arc to the truck. If the task arc is accessed by the truck, determine whether the task can be assigned to a drone. If so, randomly select a take-off and landing node pair and delete the two endpoints of task from the truck route, as shown in Figure 6.

(5) Connect drone tasks

**Figure 7:**
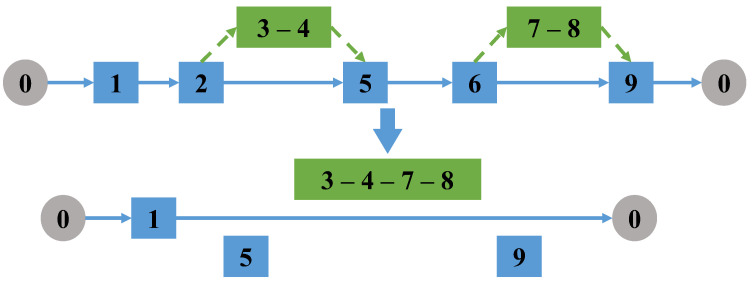
Connect the drone tasks.

Randomly select two drone task arcs that do not belong to the same linked task, then link all arcs in these two drone tasks to form a new linked task; the optimal direction and order of connecting all arcs is computed using the backtracking method. Then, delete the pair of take-off and landing nodes of the selected two tasks in the truck route, as shown in Figure 7.

(6) Destroy and reconnect drone tasks

**Figure 8:**
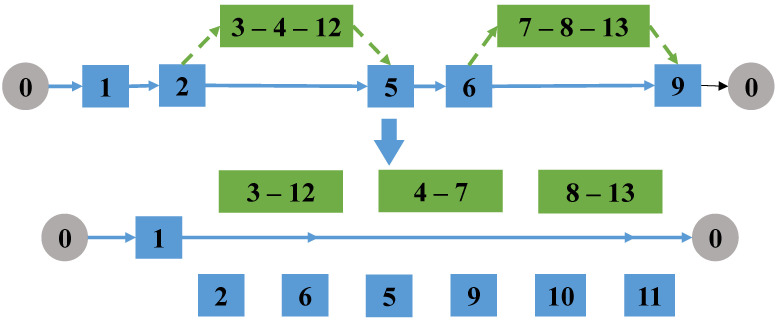
Destroy and reconnect the drone tasks.

Randomly select two drone tasks that do not belong to the same linked mission; if there is a linked task in the selected two tasks, break the connection of the linked task, and combine the two selected missions into a new linked mission. The remaining task edges from the original two tasks form one or two new tasks. Select take-off and landing points pairs for all the new tasks. Then delete the pairs of take-off and landing nodes of the original two drone tasks in the truck path, as shown in Figure 8.

#### 4.2.2. Repair Operator

**Figure 9:**
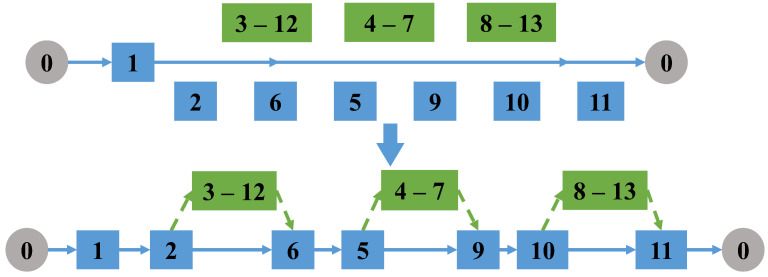
Repair operator.

The operation of the repair operator mainly includes reinserting the task into the truck route after the operation of the destruction operator, as shown in Figure 9. In this paper, one repair operator is designed: the take-off and landing point pairs (or endpoints of the task arc) generated by the destruction operator are randomly inserted into the truck route.

## 5. Simulation Experiments and Discussion

### 5.1. Parameter Setup

This article presents the LNS-SA method to address the A-VD-ARP. Because there is no universal dataset for this problem, we randomly generated three road networks, C1, C2, and C3 for A-VD-ARP. We tested the performance of the proposed algorithm through simulation experiments and analyzed the results. Finally, a real case was conducted to verify the efficiency of the algorithm. These experiments were performed on a computer with an Intel i7-10700F CPU and 32.0 GB RAM.

The information of the three datasets C1, C2, C3 is shown in Table 2. Each dataset contains three different scales of target arcs, and three computational instances (marked as A, B, and C) with the same scale are randomly generated. To generate the asymmetric road network, we selected some arcs to make them unidirectional without causing loops.

The parameter configurations are shown as follows. The maximum number of iterations is set to 1000. The initial temperature and termination temperature are set to 100 and 0.1, respectively. Besides, the parameters of the truck and drones are set according to typical situations in practical applications [18], as shown in Table 3.

### 5.2. Experiments and Analyses

To verify the effectiveness of the proposed LNS-SA, we compare it with SA, VNS, and TL. We ran each algorithm 10 times and took the average results. Table 4, Table 5 and Table 6 show the average performance of LNS-SA and the other three comparison algorithms in 27 instances. “Mean(h)” and “CPU Time(s)” are the average of 10 running results of the total patrol time and CPU running time, respectively. For each instance, the mean value of the results obtained by the four algorithms is compared, and the best solution is shown in bold.

In Table 4, Table 5 and Table 6, it can be observed that the average of the results obtained by the LNS-SA algorithm is better than the other three comparison algorithms. For C1 instances, the proposed LNS-SA can reduce the total time by 5.7% to 7.2%. For C2 instances, compared to the other three algorithms, the total time can be reduced from 1.5% to 3.2% by the LNS-SA. For C3 instances, the total time is reduced by 2.2% to 4.8%. Moreover, the overall result of the VNS algorithm is the worst. That is because the perturbation mechanism in VNS affects the convergence of the solution. The LNS could select the neighborhood search strategies randomly, which expands the search space of the algorithm and increases the possibility of converging to a better solution. As the scale of the instances increases, the total time and the CPU time increase, the LNS-SA retains its superiority.

**Figure 10:**
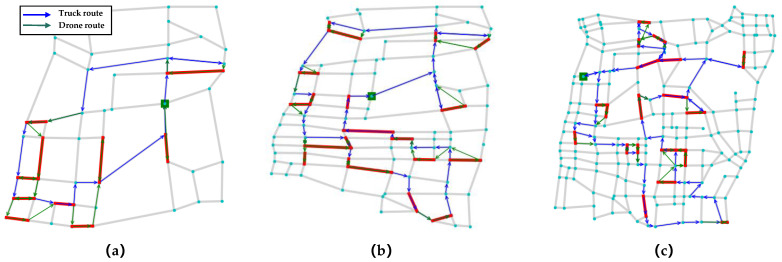
Schematic diagrams of route planning results in different instances with a different number of target edges. (**a**) E1-A; (**b**) E2-A; (**c**) E3-A.

We take the C1_E1_A, C2_E1_A, C3_E1_A as representative instances to show the result obtained by LNS-SA, as shown in Figure 10; the bold red line indicates the task arc, the blue line represents the truck route, the green line represents the drones’ route, and the green square labels the depot.

### 5.3. Sensitivity Analysis

**Figure 11:**
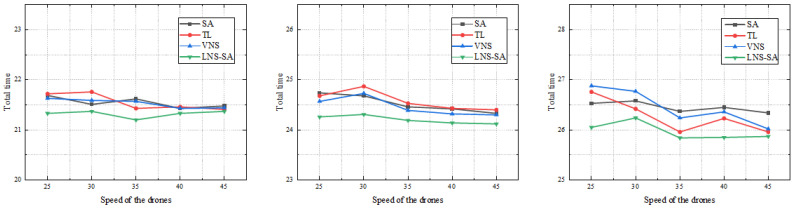
Comparison of results at different drone speeds.

To verify the influence of the drones’ speed on the experimental results, the following experiments were designed. This part of the experiment was based on three instances of different scales in the road network scenario C2, namely C2_E1_A, C2_E2_A and C2_E3_A. As with all the experiments above, each example was run 10 times and we took the average results. The speed of the drones was selected in the range of 25 km/h to 45 km/h, and a value was taken at every 5 km/h interval for comparative experiments. The experimental results are shown in Table 7 and Figure 11, respectively.

It can be seen from Figure 11 that with the increase of the speed of the drones, the total time to complete all the patrol tasks presents a downward trend. When the speed of the drone increases from 25 km/h to 45 km/h, the total patrol time decreased by 0.2–0.4 h. The total time does not decrease obviously, mainly because that the scale of the instances is large; the total time to complete the patrol mainly depends on the speed of the truck.

### 5.4. Comparison of the Algorithms with Different Numbers of Drones

**Figure 12:**
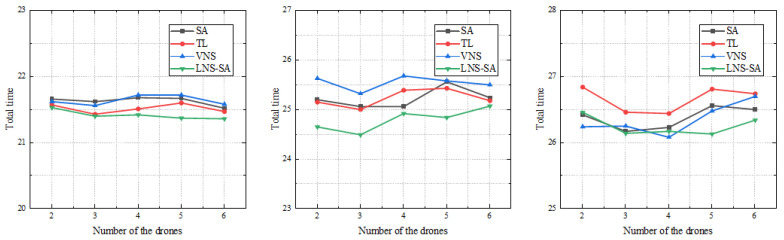
Comparison of results with different drone numbers.

Three instances of different scales, C2_E1_A, C2_E2_A, and C2_E3_A, are also used to verify the influence of the number of drones carried by the truck. Each experiment was repeated 10 times and we took the average results. We increased the number of drones from 2 to 6 for comparative experiments. All experimental results are recorded in Table 8 and Figure 12.

Figure 12 presents the results of three examples of different scales. The effect of the number of drones on the performance of the algorithm is obvious. As shown in Figure 12, with the increase of the number of drones, the total patrol time decreases first, then increases slightly, and reaches the minimum value of the patrol time when the number of drones is 3. The total patrol time decreased by 0.1–0.4 h when the number of the drones increased from 2 to 3. From this result, it can be inferred that the increase in the number of drones cannot make the patrol time keep decreasing. Too many drones make the coordination between the truck and drones difficult. Specifically, limited by the scale of the instances and the maximum flight time of the drones, there can not be too many drones performing tasks at the same time, otherwise the truck will not have enough time to recover the drones, which means that the redundant drones do not play much of a role. Therefore, in this instance, the appropriate number of drones per truck is 3.

### 5.5. Experimental Analysis on a Real Case

**Figure 13:**
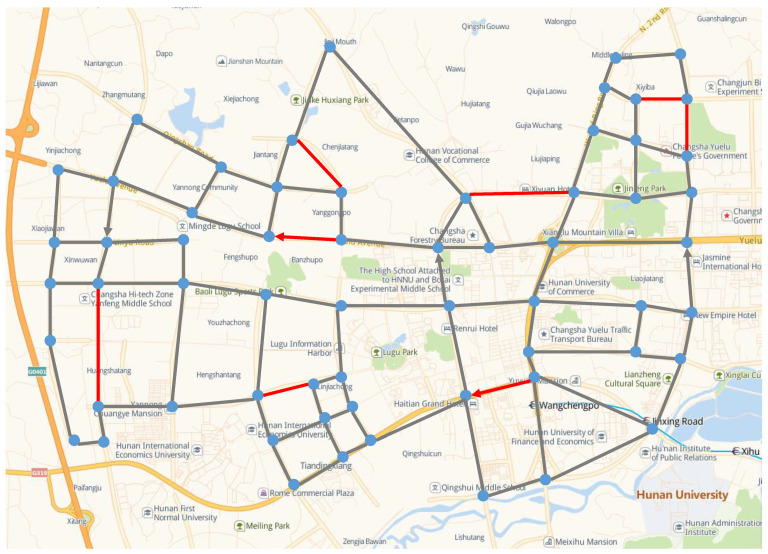
Simplified road network in a selected area of Changsha City.

In order to further verify the effectiveness of the proposed LNS-SA, a practical case based on the real world was considered. We extracted a simplified road network from Changsha China; the latitude and longitude of the location in this experiment were obtained from Google Maps, and there are 61 intersections and 94 roads in this road network. Some arcs in the road network are unidirectional, as shown in Figure 13. We increased the distance of the road by a factor of 100, making this more like a surveillance task or a city-wide patrol task.

**Figure 14:**
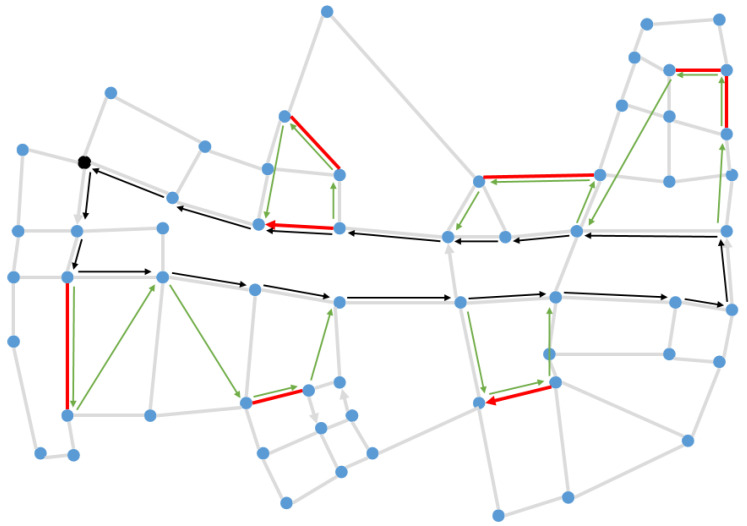
Route planning of the truck and the drones.

**Figure 15:**
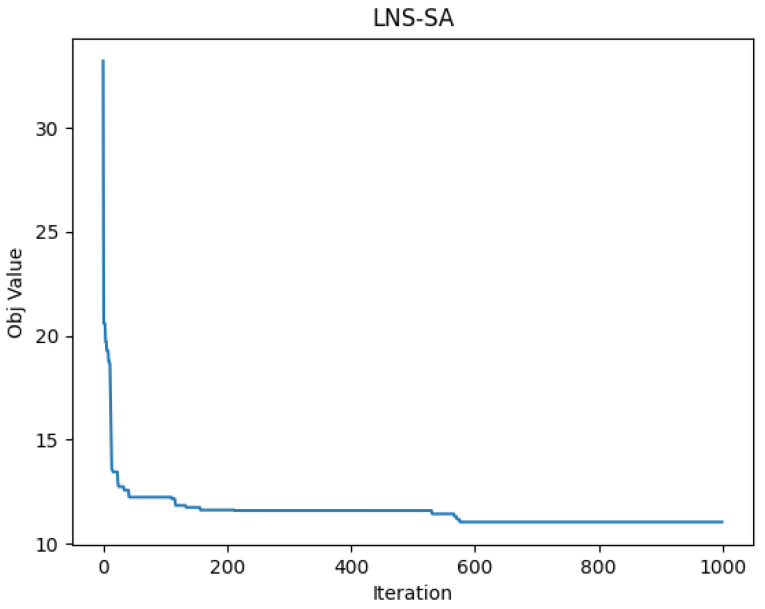
Convergence curve of the objective function value.

Figure 14 shows the patrol routes of the truck and the drones, where the bold red line represents the task arcs, the black arrow indicates the truck route, and the green arrow represents the drones’ routes. The convergence curve of the proposed LNS-SA algorithm is shown in Figure 15. The parameter settings are the same as in Section 5.1. From Figure 15, the LNS-SA generated a satisfactory solution at the 30-th iteration, demonstrating that the LNS-SA has a strong optimization capability within a short time span. The Metropolis principle can prevent the LNS-SA from prematurely converging to the local optima at the 30-th iteration. In addition, the LNS-SA allows multiple neighborhoods to search in a single solution, which expands the search space. Thus, the algorithm found a better solution at 600-th iteration.

## 6. Conclusions

In this paper, we study asymmetric arc routing by coordinating a truck and multiple drones. In this arc routing problem, a truck with multiple drones travels on an asymmetric road network—meaning some arcs of the road network are unidirectional—to perform traffic patrol tasks. The truck must travel along the road network, while the drones are not restricted to the road network. Both the truck and the drones can perform the patrol tasks. To minimize the total patrol time, a large neighborhood search algorithm with simulated annealing (LNS-SA) is proposed. Firstly, we generate the initial solution by a heuristic method. Then, six destroy operators and one repair operator are designed to iteratively optimize the solution. The simulated annealing is integrated to avoid premature convergence.

Extensive experiments were conducted to verify the effectiveness of the proposed method. Compared with other algorithms (i.e., SA, TL, and VNS) on different datasets, the LNS-SA can reduce the total patrol time by at most 7.2%. A sensitivity analysis was performed to analyze the impact of the number of drones and drone speed. As the drone number increased, the total time continued to decrease, but the rate of the descent slowed down and finally stopped decreasing. In addition, the total patrol time decreased slightly with the drone speed increasing due to the scale of the datasets. Moreover, a real case in China Changsha was conducted to verify the effectiveness of the LNS-SA.

In the future, we will further explore the coordinated vehicle-drone arc routing problem in different scenarios. The objective function in this paper only considers the total time, which is not comprehensive enough. In the next stage, we will consider the multi-objective optimization problem under more realistic conditions. We will also attempt to design more efficient operators to solve the problem.

## Figures and Tables

**Table 1:** Symbols and descriptions.

**Symbols**	**Descriptions**
G	The asymmetric road network
V	Set of road intersections, V={1,2,…,n}
$V^{-}$	Set of start node of the truck route
$V^{+}$	Set of end node of the truck route
A	$\mathrm{Set}\;\mathrm{of}\;\mathrm{arcs},\;A=\left\{a_{ij}=\left(i,j\right)|i,j\in V\right\}$
T	Set of task arcs, T⊆A
TD	Set of task arcs which can only be accessed by drones, TD⊆T
$ce_{i}$	The task of the drone, which may be a single arc or a linked arc
CE	$\mathrm{Collection}\;\mathrm{of}\;\mathrm{tasks}\;\mathrm{performed}\;\mathrm{by}\;\mathrm{drones},\;CE=\left\{ce_{1},ce_{2},\ldots ,ce_{t}\right\}$
D	Set of drones, D={1,2,…,d}
GR	$\mathrm{The}\;\mathrm{truck}\;\mathrm{route},\;\mathrm{represented}\;\mathrm{by}\;\mathrm{the}\;\mathrm{nodes}\;\mathrm{that}\;\mathrm{the}\;\mathrm{truck}\;\mathrm{visits}\;\mathrm{in}\;\mathrm{turn},\;R=\left\{v_{0},\ldots ,v_{k},\ldots ,v_{pc}\right\}$
$\langle s,ce_{i},e\rangle$	A drone route, represented by the nodes that the drone passes through in sequence. The drone is released from node s, and recovered at node e after accessing the task arc $ce_{i}$
U	$\mathrm{Set}\;\mathrm{of}\;\mathrm{drone}\;\mathrm{routes},\;U=\left\{\langle s,ce_{1},e\rangle ,\langle s,ce_{2},e\rangle ,\ldots ,\langle s,ce_{t},e\rangle \right\}$
$w_{ij}$	$\mathrm{Length}\;\mathrm{of}\;\mathrm{the}\;\mathrm{arc}\;a_{ij}$
$t_{i}$	The time that the truck arrives at node i
$t_{i}^{k}$	The time that the k-th drone arrives at node i
$t_{i}^{\prime }$	The time the truck waits for the drone after arriving at node i
$v_{g}$	The speed of the truck
$v_{d}$	The speed of the drone
P	Drones’ maximum flight time
**Decision variables**	**Description**
$x_{ij}$	$\mathrm{Binary}\;\mathrm{variable},\;x_{ij}$$=1\;\mathrm{if}\;\mathrm{the}\;\mathrm{vehicle}\;\mathrm{passes}\;\mathrm{the}\;\mathrm{edge}\;a_{ij}$; otherwise, 0
$y_{se}^{k}$	$\mathrm{Binary}\;\mathrm{variable},\;y_{se}^{d}=1$ if the d-th drone is released from node s and recovered at node e; otherwise, 0
$av_{i}^{k}$	Binary variable, auxiliary variable used to represent the state, whether the k-th drone is on the truck when the truck reaches node i$,\;\mathrm{if}\;\mathrm{the}\;\mathrm{drone}\;\mathrm{is}\;\mathrm{on}\;\mathrm{the}\;\mathrm{truck},\;av_{i}^{k}=0$, otherwise 1
$av^{t,k}$	$\mathrm{Binary}\;\mathrm{variable},\;\mathrm{auxiliary}\;\mathrm{variable}\;\mathrm{used}\;\mathrm{to}\;\mathrm{represent}\;\mathrm{the}\;\mathrm{state},\;av^{t,k}=0$ if the k-th drone is released at time t, otherwise 1

**Table 2:** The scale of the instances.

Instances	The Number of Nodes	The Number of Edges	The Number of Tasks	
C1	50	79	E1	5 (A/B/C)
			E2	10 (A/B/C)
			E3	20 (A/B/C)
C2	100	167	E1	10 (A/B/C)
			E2	20 (A/B/C)
			E3	40 (A/B/C)
C3	200	339	E1	10 (A/B/C)
			E2	20 (A/B/C)
			E3	40 (A/B/C)

**Table 3:** Experimental parameter design.

Parameters	Value
The number of drones on a truck	3
The speed of the truck	30 km/h
The speed of the drones	35 km/h
The maximum flight time of the drones	0.67 h

**Table 4:** Algorithm Performance on C1 Instances.

No.	Instances	|V|	|A|	|T|	SA		TL		VNS		LNS-SA	
					Result	CPU Time	Result	CPU Time	Result	CPU Time	Result	CPU Time
1	C1_E1_A	50	79	5	11.1154	36.24	10.7185	32.65	11.04	35.79	**10.0714**	41.46
2	C1_E1_B	50	79	5	10.6915	32.25	10.8601	31.04	12.0373	34.51	**10.665**	38.52
3	C1_E1_C	50	79	5	12.7155	29.74	12.4968	29.56	12.3414	30.86	**11.8067**	38.31
4	C1_E2_A	50	79	10	16.8716	46.12	15.2329	45.17	16.4487	43.82	**14.5617**	52.05
5	C1_E2_B	50	79	10	12.6276	41.85	12.38	43.25	12.5059	43.58	**12.1306**	52.17
6	C1_E2_C	50	79	10	12.0499	44.19	12.0605	43.33	11.9189	46.25	**11.5593**	55.66
7	C1_E3_A	50	79	20	14.8979	54.1	13.7787	52.69	14.2644	57.22	**13.3425**	53.16
8	C1_E3_B	50	79	20	16.9169	48.01	16.8789	49.53	16.8885	50.52	**15.3091**	50.18
9	C1_E3_C	50	79	20	16.5547	47.87	17.2401	46.09	17.7648	47.94	**16.4684**	53.45
GAP (%)					7.2%		5.7%		6.9%		-	

**Table 5:** Algorithm Performance on C2 Instances.

No.	Instances	|V|	|A|	|T|	SA		TL		VNS		LNS-SA	
					Result	CPU Time	Result	CPU Time	Result	CPU Time	Result	CPU Time
10	C2_E1_A	100	167	10	21.782	57.45	21.5014	54.97	21.8286	55.9	**21.1996**	60.41
11	C2_E1_B	100	167	10	19.636	53.09	19.7822	50.39	19.6207	51.54	**19.078**	55.92
12	C2_E1_C	100	167	10	20.2279	54.27	19.9678	55.5	19.9193	56.09	**19.7367**	68.97
13	C2_E2_A	100	167	20	**22.9637**	76.92	24.258	84.9	24.291	76.76	24.0491	99.61
14	C2_E2_B	100	167	20	**25.8607**	77.77	26.2442	72.53	26.0515	71.07	25.9661	78.79
15	C2_E2_C	100	167	20	25.7421	90.79	24.6352	85.6	25.4427	88.97	**24.3107**	96.55
16	C2_E3_A	100	167	40	**25.6765**	102.3	26.7669	97.01	28.392	103.22	25.8969	103.86
17	C2_E3_B	100	167	40	28.392	88.65	28.3228	86.43	27.7454	87.15	**27.2731**	93.5
18	C2_E3_C	100	167	40	28.9166	101.27	30.3552	98.56	29.7808	98.68	**28.4415**	101.77
GAP (%)					1.5%		2.6%		3.2%		-	

**Table 6:** Algorithm Performance on C3 Instances.

No.	Instances	|V|	|A|	|T|	SA		TL		VNS		LNS-SA	
					Result	CPU Time	Result	CPU Time	Result	CPU Time	Result	CPU Time
19	C3_E1_A	200	339	10	28.74	53.847	29.156	51.17	28.4569	50.707	**28.345**	57.937
20	C3_E1_B	200	339	10	27.974	52.347	30.225	50.17	27.6077	50.697	**26.751**	56.717
21	C3_E1_C	200	339	10	31.053	58.447	33.594	57.67	36.6992	61.317	**30.757**	66.077
22	C3_E2_A	200	339	20	33.560	77.287	36.241	77.15	36.7257	77.637	**33.200**	77.597
23	C3_E2_B	200	339	20	35.072	78.347	35.706	73.19	35.0856	78.967	**34.616**	78.797
24	C3_E2_C	200	339	20	36.38	94.797	36.068	86.94	35.5344	90.897	**35.371**	94.107
25	C3_E3_A	200	339	40	35.266	106.99	36.052	98.78	**35.2449**	100.56	37.228	100.15
26	C3_E3_B	200	339	40	41.601	113.69	39.36	109.17	41.6981	111.68	**38.475**	111.57
27	C3_E3_C	200	339	40	41.928	112.76	41.78	109.60	41.3439	113.07	**39.865**	110.78
GAP (%)					2.2%		4.8%		4.6%		-	

**Table 7:** Experimental results with different drone speeds.

Instances	Algorithms	Speed of the Drones				
		25	30	35	40	45
C2_E1_A	SA	21.69	21.51	21.62	21.43	21.48
	TL	21.72	21.76	21.43	21.46	21.41
	VNS	21.63	21.59	21.57	21.43	21.44
	LNS-SA	21.33	21.37	21.2	21.33	21.37
C2_E2_A	SA	24.74	24.68	24.46	24.42	24.33
	TL	24.68	24.87	24.53	24.43	24.4
	VNS	24.57	24.73	24.39	24.32	24.3
	LNS-SA	24.26	24.31	24.19	24.14	24.12
C2_E3_A	SA	26.53	26.58	26.37	26.45	26.34
	TL	26.76	26.42	25.96	26.23	25.96
	VNS	26.88	26.77	26.24	26.36	26.02
	LNS-SA	26.05	26.24	25.84	25.85	25.87

**Table 8:** Experimental results with different drone numbers.

Instances	Algorithms	Number of Drones				
		2	3	4	5	6
C2_E1_A	SA	21.66	21.62	21.68	21.67	21.52
	TL	21.57	21.43	21.51	21.6	21.47
	VNS	21.62	21.56	21.72	21.72	21.58
	LNS-SA	21.53	21.4	21.42	21.37	21.36
C2_E2_A	SA	25.2	25.06	25.06	25.56	25.24
	TL	25.15	25.00	25.39	25.43	25.18
	VNS	25.63	25.32	25.68	25.58	25.50
	LNS-SA	24.65	24.49	24.92	24.84	25.07
C2_E3_A	SA	26.42	26.17	26.23	26.56	26.5
	TL	26.84	26.46	26.44	26.81	26.74
	VNS	26.24	26.25	26.08	26.48	26.7
	LNS-SA	26.46	26.14	26.17	26.13	26.34

## Data Availability

Not applicable.
